# Long-Term Plasticity Is Proportional to Theta-Activity

**DOI:** 10.1371/journal.pone.0005850

**Published:** 2009-06-09

**Authors:** Marian Tsanov, Denise Manahan-Vaughan

**Affiliations:** 1 Department of Experimental Neurophysiology, Medical Faculty, Ruhr University Bochum, Bochum, Germany; 2 International Graduate School for Neuroscience, Ruhr University Bochum, Bochum, Germany; Tel Aviv University, Israel

## Abstract

**Background:**

Theta rhythm in the hippocampal formation is a main feature of exploratory behaviour and is believed to enable the encoding of new spatial information and the modification of synaptic weights. Cyclic changes of dentate gyrus excitability during theta rhythm are related to its function, but whether theta epochs *per se* are able to alter network properties of dentate gyrus for long time-periods is still poorly understood.

**Methodology/Principal Findings:**

We used low-frequency stimulation protocols that amplify the power of endogenous theta oscillations, in order to estimate the plasticity effect of endogenous theta oscillations on a population level. We found that stimulation-induced augmentation of the theta rhythm is linked to a subsequent increase of neuronal excitability and decrease of the synaptic response. This EPSP-to-Spike uncoupling is related to an increased postsynaptic spiking on the positive phases of theta frequency oscillations. Parallel increase of the field EPSP slope and the population spike occurs only after concurrent pre- and postsynaptic activation. Furthermore, we observed that long-term potentiation (>24 h) occurs in the dentate gyrus of freely behaving adult rats after phasic activity of entorhinal afferents in the theta-frequency range. This plasticity is proportional to the field bursting activity of granule cells during the stimulation, and may comprise a key step in spatial information transfer. Long-term potentiation of the synaptic component occurs only when the afferent stimulus precedes the evoked population burst, and is input-specific.

**Conclusions/Significance:**

Our data confirm the role of the dentate gyrus in filtering information to the subsequent network during the activated state of the hippocampus.

## Introduction

In an effort to more closely approximate endogenous conditions for information storage in the hippocampus, many studies have addressed the relationship between synaptic plasticity and neuronal oscillations during exploratory behaviour. Long-term potentiation (LTP) of synaptic strength is widely supported as a spatial memory mechanism. However, the network conditions under which LTP is initiated during exploration are not yet clear. Field potential oscillations at theta frequency (5–12 Hz) are believed critical for the acquisition of new information by active hippocampal ensembles [1], and are in fact a typical feature of network activity during novel spatial exploration. Modification of synaptic weights happens very fast during the activated state of the hippocampus [2], with entorhinal-hippocampal network oscillations at theta frequency playing a crucial role in this process [3]. Thus, coherent oscillations of cell assemblies during theta activity offer a possible mechanism for temporal coding and decoding where segregated assembles can “wire together” and store information among linked neurons in a phase-locked manner. Neocortico-hippocampal transfer of new spatial information occurs during exploratory behaviour. If simultaneous firing of hippocampal neurons during explorative-learning behaviour is sufficient for *temporary* synaptic modification, then the question arises as to whether similar cooperative firing can trigger persistent alterations in synaptic strength.

Hippocampal place cells are either silent, or discharge with single spikes, during behavioural arousal [4]. Complex spike bursts are also known to occur [5], [6], in the time course of the depolarizing theta oscillation, due to the rhythmic decrease of perisomatic inhibition [3]. These spike bursts significantly increase the effectiveness of synaptic transmission, and at the same time, may induce synaptic plasticity [7], [8], [9]. Pairing presynaptic activity with postsynaptic bursts in hippocampal pyramidal cells in vitro results in LTP of activated synapses [10], [11]. Similarly, a long-lasting enhancement of the field excitatory postsynaptic potential (fEPSP) is elicited, if hippocampal afferents are stimulated synchronously on the peak of theta oscillations, and LTP is more effectively induced in the dentate gyrus when tetanisation is delivered on the positive phases of theta frequency oscillations in anaesthetised animals [12], [13]. During naturally occurring, locomotion-induced theta oscillations, LTP is also preferentially induced by stimuli delivered near the local theta peak in behaving animals [12]. This rhythm is naturally prominent in the hippocampus during exploratory behaviour and can be subdivided into high theta (8–12 Hz), which is dependent on motor activity [14], [15], [16], and low theta (5–8 Hz), which is triggered by medial septum activation [14], [17]. LTP can be induced in the time course of both locomotion- [12], [18] and cholinergic- induced [19], [20] theta. The synaptic change initiated during locomotion activity endures for at least 48 h in the awake rat [12], consistent with a role in long-term information storage. Strikingly, tetanic stimulation at the *trough* of theta, induces LTD in the CA1 region of freely moving rats, indicating that theta episode-related plasticity is bidirectional [18]. Studies using stimulation protocols using theta frequency in vitro [21] and in vivo [22], [23] led to the proposal that hippocampal theta rhythm promotes both the induction of LTP and its subsequent reversal. An open question however, is whether bidirectional synaptic plasticity during theta episodes can result, not only from external tetanic stimulation under controlled experimental conditions, but also from intrinsic neuronal bursts reflecting the naturalistic state, and to what extent this plasticity is related to the firing history of hippocampal neurons. Here we test the hypothesis that physiological changes of hippocampal spectral power enable selective long-term alteration of the synaptic and excitatory properties of the dentate gyrus granule cells.

The dentate gyrus acts as a major informational gateway to the hippocampus and plays both an anatomical and a physiological role in filtering information to, and modifying synaptic connectivity in, the CA3 network, where spatial information is assumed to be temporarily held [2], [24], [25]. Reciprocally-connected dentate gyrus and CA3 networks enable the encoding of specific information in context [26], [27] and accurate recall of sequences [24]. Synchronous activity of a subset of granule cells in the dentate gyrus is sufficient to distinguish small changes in sensory input during rate remapping in CA3 [28]. If conjoint firing of neurons during explorative-learning behaviour is a sufficient condition for synaptic modification [29], [30], then this suggests that similar cooperative firing may correlate with long-term alterations in synaptic weights. In the present study, we examined the possibility that dentate gyrus granule cells may change their firing patterns proportionally to the preceding spiking activity in order to enable synaptic information storage. We used low-frequency stimulation protocols to amplify the power of endogenous theta oscillations in the dentate gyrus and to examine the consequences on synaptic strength. Our protocol of brief theta frequency stimulation was able to persistently raise theta spectral power in a certain percentage of the stimulated animals. In these cases an increase of the population spike (PS) was paralleled by a decrease of fEPSP slope. In addition, we found that LTP occurs after phasic activation of entorhinal afferents in the theta-frequency range and the efficacy of signal transmission through dentate region was proportional to the field bursting activity of granule cells during the stimulation. Our data support the hypothesis that the dentate gyrus is actively involved in increasing the effectiveness of synaptic transmission, and also redistributes the sign of synaptic plasticity towards potentiation or depression, depending on the entorhinal input.

## Results

### Brief 8 Hz entorhinal stimulation leads to theta rhythm augmentation in the dentate gyrus

**Figure 1:**
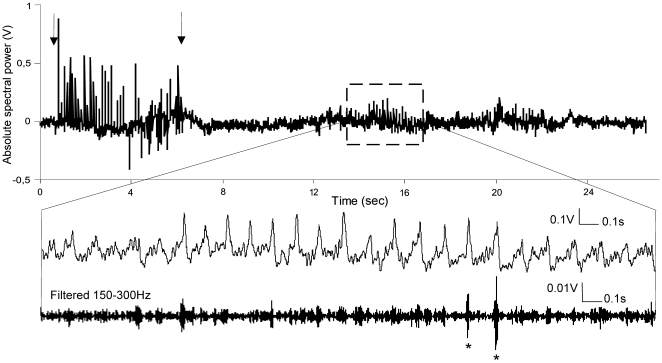
8 Hz stimulation-induced theta oscillations. Sample EEG trace from the dentate gyrus representing 8 Hz (30 pulses) stimulation of the medial perforant path and subsequent intrinsic theta oscillations. Arrows indicate the start and the end of the stimulation. The increased theta activity, after the stimulation, is highlighted in the sample trace below. Application of 150–300 Hz band-pass filter reveals ripple oscillations on the positive peak of theta cycle, marked with asterisks (bottom trace).

Cyclic excitation of dentate gyrus cells at 8 Hz, by means of medial perforant path stimulation in freely behaving adult rats, was expected to induce synchronization of neuronal populations at this frequency. In order to prevent a generalized (epileptiform) response for the whole dentate network, we restricted the duration of the stimulation protocol to 30 pulses (Fig. 1). Intrahippocampal EEG was recorded, and extracellular somatic responses were measured throughout the baseline period, stimulation protocol and during the subsequent 4 h.

The weak 8 Hz protocol (30 pulses) induced an alteration of the neuronal firing only in a subset of the used animals. By comparing the synaptic changes of the neuronal populations that underwent certain firing patterns to those that were not affected by the stimulation, we were able to link the local field oscillations with subsequent synaptic plasticity. The stimulation rate in our experiments was related to the observation that during novelty exploration the most robust elevation of low-frequency spectral power in the dentate gyrus is close to 8 Hz.

**Figure 2:**
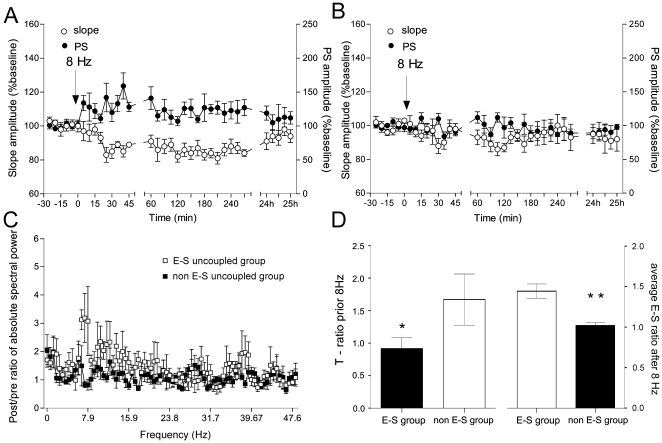
Theta oscillation-dependent E-S uncoupling. (A) Brief 8 Hz (30 pulse) stimulation of the perforant pathway induced, in 43% of all cases, a decrease of the fEPSP slope and an increase of PS amplitude, referred to as EPSP- to-spike uncoupling (E-S uncoupled group, *n* = 6). (B) A lack of change in fEPSP and PS was observed in the remaining 57% of animals (non E-S uncoupled group, *n* = 6). (C) An increase of theta spectral power for 18 sec after the stimulation, compared with the EEG epochs of 18 sec prior to the stimulation, was observed in the E-S group only (white squares) (student's paired *t* test, *p*<0.01, *n* = 6). The non-E-S group (black squares) did not respond with increased theta. (D) Theta ratio (T-ratio), representing the absolute values of theta range (5–10 Hz) divided by the absolute values of delta range (2–4 Hz), in both groups prior to the stimulation, is shown in the left panel. The right panel demonstrates the difference in the E-S uncoupling ratio (summary values from 4 h of recordings of the ratio between PS amplitude and EPSP slope) from the same animals, (* *p*<0.05).

Comparing the field alterations to potentials evoked during the baseline period, the fEPSP responses were classified in two groups: a group that displayed an increase of the PS combined with a parallel decrease of the fEPSP slope, and a group that displayed no significant changes in these parameters. The first group, referred to as the “EPSP-to-spike uncoupled group” consisted of 43% of all cases (Fig. 2*A*), whereas the weakly affected, or “non-E-S uncoupled group” comprised the remaining 57% (Fig. 2*B*). The small, but long-term and significant, increases in PS paralleled by decreases in fEPSP, compared to baseline values (ANOVA, *p*<0.01, *n* = 6) support the possibility that that the E-S ratio reflects excitability-related plasticity.

We analyzed the EEG spectral changes in a time window of 18 sec after the stimulation and detected augmented theta rhythm episodes in the E-S uncoupled group only (Fig. 1). A significant enhancement of theta frequency power was observed in the E-S uncoupled group (ANOVA, *p*<0.01, *n* = 6) compared to the spectral values of non-E-S uncoupled group (ANOVA, *p*>0.05, *n* = 6), (Fig. 2C). Filtered in the range of 150–300 Hz, the EEG profile of the theta epochs in the E-S uncoupled group revealed occasionally multiple spiking events superimposed on the positive phases of theta (Fig. 1).

An intriguing issue was why the same stimulation protocol evokes E-S uncoupling only in certain cases. We hypothesized that external 8 Hz stimulation interferes with natural theta rhythms in the dentate gyrus, resulting in antagonism of the modulation of synaptic and intrinsic neuronal properties. For this purpose we compared the theta activity in both groups, as measured by the ratio between the absolute values of theta and delta spectral powers (T-ratio) for 18 sec prior to the stimulation. We found that both groups differ in their theta activity before the stimulation protocol (Fig. 2D). The E-S uncoupled group was characterized by a lower T-ratio, demonstrating that fEPSP changes, related to increased theta power, tend to occur when the 8 Hz stimulation is given during periods with low theta amplitude.

The theta epochs, that characterized the E-S uncoupled group, were recorded *after* the initial 8 Hz stimulation. Thus, the spiking episodes were not paired with stimulus-evoked entorhinal activity. The depression of the fEPSP seen, is in line with the observation that inactive synapses, during membrane depolarization by a converging synaptic input, undergo heterosynaptic LTD of the fEPSP [41]. Furthermore, E-S potentiation has been consistently associated with heterosynaptic E-S depression, that may serve to enhance input specificity [42], [43]. To test the hypothesis that increased spiking frequency, when paired with input-specific efferent activity, will result in an increased response to this input, our next step was to combine test-pulse stimulation with stimulus-induced theta activity, and to measure their relationship.

### Stimulation-induced theta oscillations in the time course of a 2.5 Hz frequency protocol

As our goal was to observe theta augmentation in the time-course of the long enough stimulation protocol, we decided to stimulate medial perforant path with 2.5 Hz (900 pulses). By this means, the external stimuli and the endogenous cyclic theta depolarization (the frequency of which was about 8 Hz in our animals) will not interfere in the same phase, and thus the probability for generalized depolarization of the dentate gyrus in the course of the 900 pulses is reduced to a minimum.

**Figure 3:**
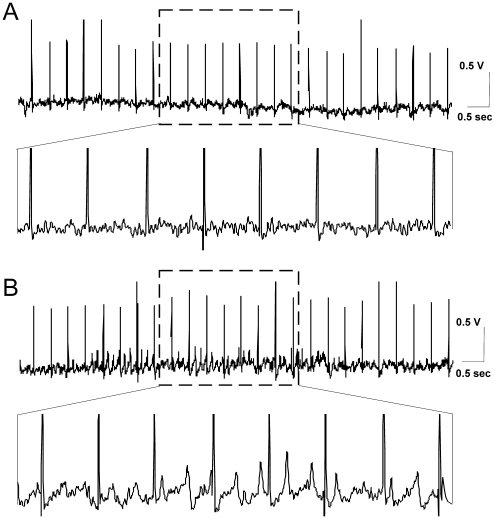
Stimulation-induced theta oscillations throughout 2.5 Hz protocol. (A) Sample EEG trace, in the time course of 2.5 Hz stimulation, from an animal that did not respond with subsequent fEPSP changes to the stimulation protocol. (B) Sample EEG trace, in the time course of 2.5 Hz stimulation, from an animal that displayed E-S uncoupling after the stimulation protocol. The amplified EEG epoch, demonstrates an occurrence of field patterns of high amplitude in the theta range, from the same EEG record.

**Figure 4:**
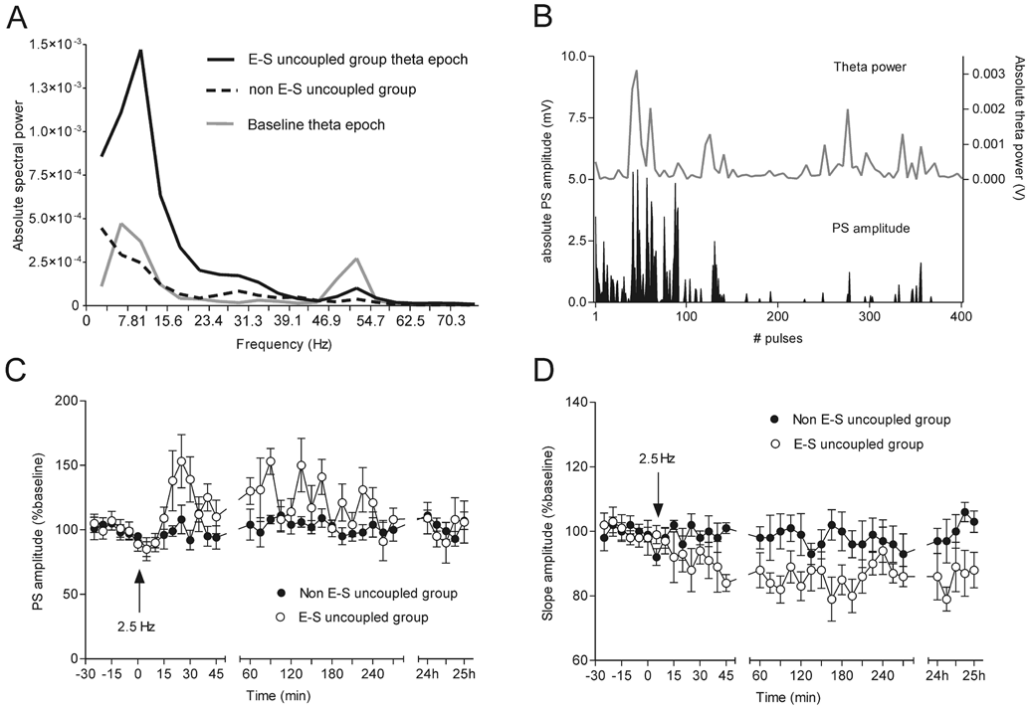
2.5 Hz stimulation-induced theta augmentation is linked to subsequent fEPSP changes. (A) A robust increase of oscillations in theta range, with a peak at 8–12 Hz, is observed in the E-S uncoupled group (*n* = 12) (black trace) during 2.5 Hz stimulation. The grey trace represents baseline theta (n = 6). Note the higher oscillatory power of the gamma range in the control group. The standard errors of the data are not shown for better visualisation of the graph. A significant difference (student's paired *t* test, *p*<0.01) between the E-S uncoupled group and baseline theta values, was detected in the frequencies between 8 and 15 Hz, as well as between 50 and 55 Hz. The non-E-S uncoupled group (interrupted trace) is characterized by a low spectral power (*n* = 6) with highest values occurring between 2 and 4 Hz. (B) Comparison of the fluctuations of PS amplitude and theta power values throughout the stimulation. This example represents a case of the E-S uncoupled group, demonstrating the absolute values of both parameters during the first 400 pulses. Note the concurrent increase of granule cell excitability and theta spectral power. (C) Population spike amplitude in the cases with no generalized response to 2.5 Hz (900 pulse) can be subdivided into a group that displayed increased PS amplitude (white circles, n = 6) and a group that displayed no significant changes (filled circles, *n* = 6). (D) Field EPSP slope measurements of the same animals reveal behaviour that is opposite to the PS response. Compared to the baseline period, the fEPSP, in the affected cases, developed a long-term decrease (E-S uncoupling).

We subdivided the recordings that displayed no generalized dentate depolarization, into two groups, depending on whether a persistent change in the oscillatory activity occurred, in comparison to the baseline period. For one group of animals 2.5 Hz stimulation did not evoke theta rhythm augmentation (Fig. 3A), whereas the other group of animals, comprised the cases where in the course of 900 pulses a robust increase of theta oscillation occurred (Fig. 3B). Spectral power analysis of the EEG epochs between the test-pulses revealed that the major difference of the spectral power is in the theta range (Fig. 4A, B). This increase was much higher than the baseline theta epochs, suggesting that the stimulation protocol induced a much more potent population response in this frequency range (Fig. 4A) (student's paired *t* test, *p*<0.001, *n* = 8). In comparison to the baseline theta peak (around 8 Hz), a slight shift towards higher theta was observed throughout the stimulation (around 12 Hz). Parallel changes in the gamma power (particularly 45–60 Hz) were also detected for the compared groups, but they were less apparent when compared to the theta range (Fig. 4A). As was the case with the 8 Hz stimulation protocol, we detected an increase of the PS (Fig. 4C, ANOVA, *p*<0.01, *n* = 6) and a parallel decrease of the fEPSP slope (Fig. 4D, ANOVA, *p*<0.01, *n* = 6) in the cases with augmented theta spectral power (E-S uncoupled group), but not in the rest of the cases (non E-S uncoupled group).

**Figure 5:**
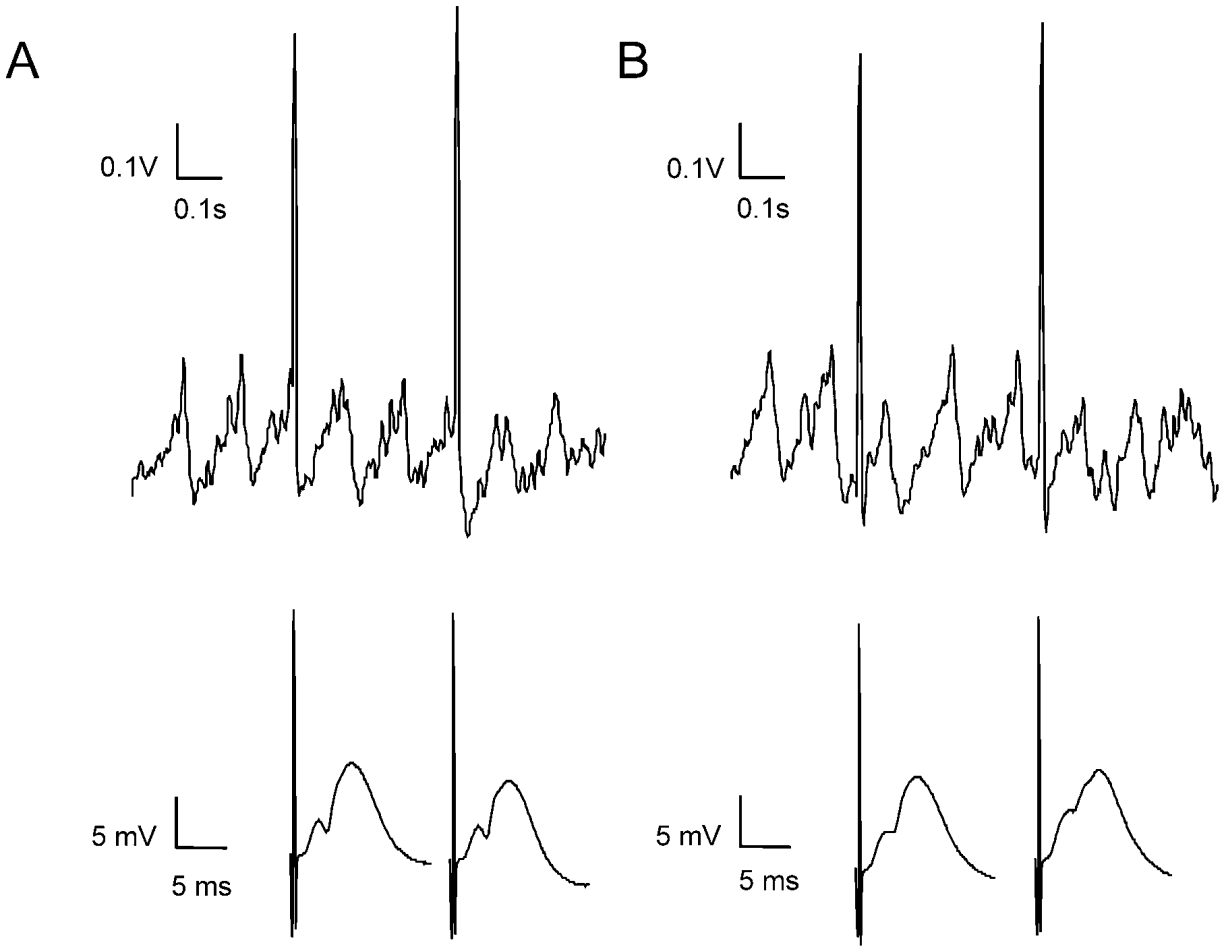
fEPSP profile depends on the timing of the stimulation-induced theta phase. (A) Sample EEG trace from a case with stimulation-induced theta oscillations, where positive peaks coincide with entorhinal stimuli (upper trace). The same field potentials represented in ms time scale (bottom traces) show a clear population spike. (B) Sample EEG trace from a case with stimulation-induced theta oscillations, where the positive peaks do not coincide with the entorhinal stimuli (upper trace). The population spike of the same field potentials, measured at ms time scale (bottom traces) is almost absent.

Our next goal was to examine if the E-S uncoupling occurs because of a temporal dissociation between the increased excitability on the positive peak of theta and the timing of the efferent entorhinal spike. First, we investigated if increased theta epochs are related to increased excitability. For this purpose we compared the population spike (PS) amplitude throughout the stimulation with the EEG spectral power and particularly its theta range (Fig. 4B). We detected a significant correlation between the absolute theta values and the mean of the PS amplitude (Pearsons, *r* = 0.506, *p*<0.001, *n* = 4). In order to relate dentate gyrus excitability to the theta phase, we compared the fEPSP in cases where the entorhinal spike was superimposed on the positive peak of theta (Fig. 5A), to cases where the entorhinal spike and the positive peak had more than 40 ms proximity (Fig. 5B). The amplitude of dentate gyrus PS was predominantly higher in the cases when both events coincided in time. In cases where the stimulus was out-of-phase stimulus, a tendency towards lower granule cell field responses was evident. As most of the spikes during the 2.5 Hz (900 pulses) stimulation were not superimposed on the theta peaks, the final outcome was an absence of potentiation, and a depression of the fEPSP (Fig. 4C, D). Similarly, during brief 8 Hz (30 pulse) stimulation, the theta augmented epochs also occurred in the absence of any entorhinal input, which led to a depression of the fEPSP, although the stimulation-induced spikes resulted in an increased excitability, as reflected by an increase in PS amplitude.

We were therefore interested to examine whether fEPSP potentiation will occur if *every* entorhinal spike coincides temporally with a field depolarization event. For this purpose, we measured long-term changes of the fEPSP, in cases of generalized dentate gyrus activation where the endogenous depolarization was in phase with the entorhinal stimuli.

### Theta frequency-induced field burst firing in the dentate gyrus

**Figure 6:**
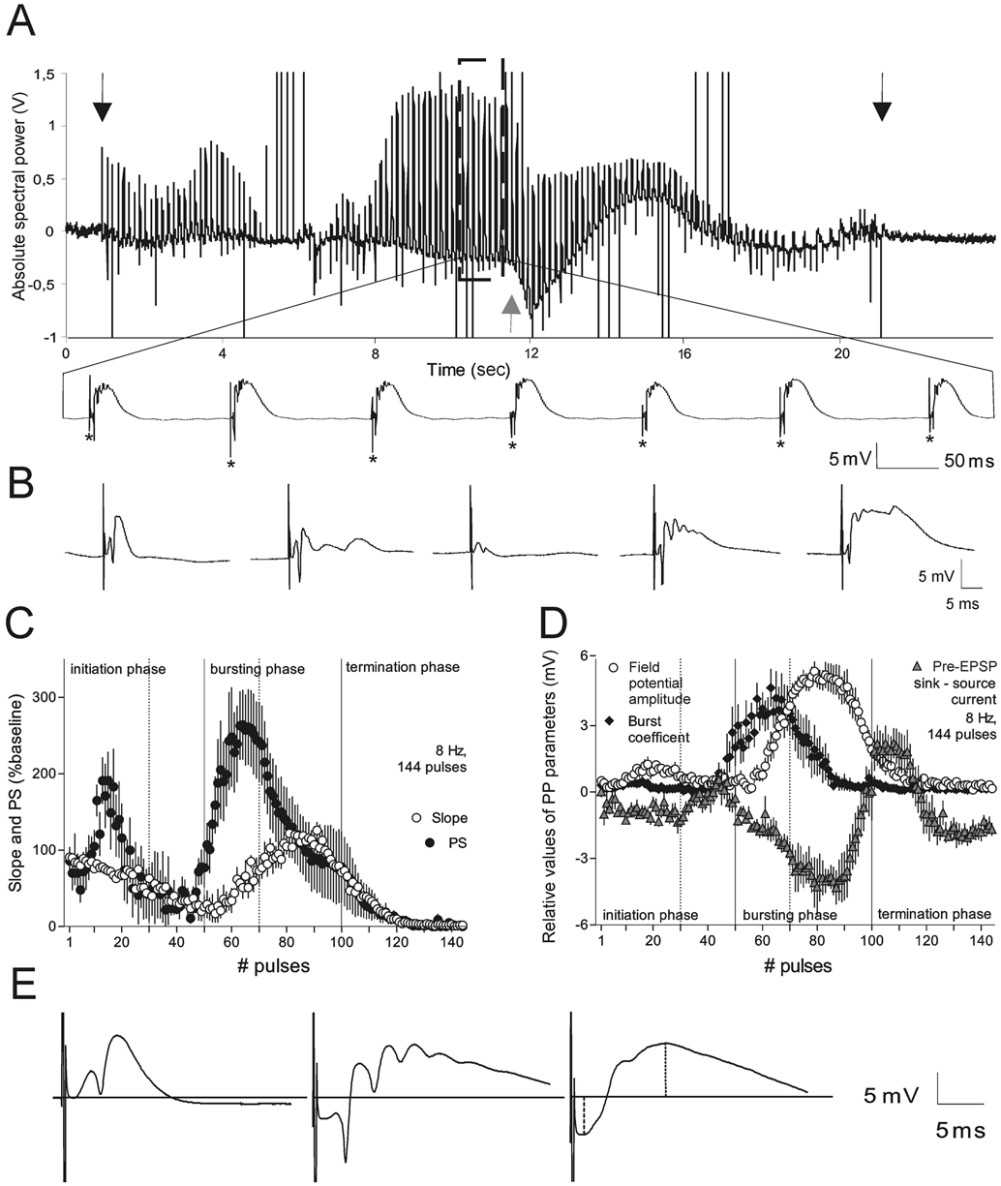
Electrophysiological properties of the dentate gyrus granule cell field response to 8 Hz stimulation. (A) Sample intrahippocampal electroencephalogram (EEG) demonstrates the appearance of field bursts. Arrows indicate the start and the end of perforant path 8 Hz stimulation; grey arrow shows the appearance of negative shift of DC-potential. Bottom sample trace amplifies the field responses during the bursting phase. Asterisks show the test-pulses that precede each fEPSP bursting response. (B) Sample recordings, from left to right, represent fEPSP traces in controls: early initiation phase, late initiation phase, early bursting phase and late bursting phase, respectively. (C) Summary data show population spike (PS) amplitude and fEPSP slope changes in the course of 8 Hz (144 pulse) stimulation (n = 5). The main phases (initiation, bursting and termination) are divided by vertical lines; the dotted lines indicate the subdivision of the phases (early and late parts). (D) fEPSP parameters involved in the calculation of plasticity factor (PF, see methods) in the time course of 8 Hz stimulation (*n* = 5). (E) Sample recordings from baseline period and the early and late bursting phases, respectively. The distance between fEPSP initiation and the horizontal line, normalized to the baseline, shows the amplitude of the extracellular pre-EPSP current sink (lower dotted line). The highest value of the fEPSP, normalized to the baseline (upper dotted line), reflects the positive field potential amplitude (PA).

To test the hypothesis that increased spiking, paired with input-specific efferent activity, correlates with the degree of LTP at this input, our first goal was to induce intrinsic field bursting at the theta frequency, and to measure its long-term effect on the synaptic response. Here, we used 8 Hz stimulation, because the spectral power of network activity recorded from dentate gyrus granule cells during exploratory behaviour was found to be exactly in the range of 8 Hz (Fig. 4A). As the maximal excitability and bursting activity during 8 Hz stimulation typically occurred in the period between the 50^th^ and 75^th^ pulse, with no cases occurring after the 144^th^ pulse we gave 8 Hz stimulation 144 times (Fig. 6).

Stimulation at 8 Hz induced pulse-evoked synchronised discharges in the theta range (Fig. 6A). During the stimulation, generalized field burst firing was preceded and followed by patterned changes in the fEPSP (Fig. 6B). In relation to these changes we identified 3 main phases: (1) an initiation (2) a bursting and (3) a termination phase (Fig. 6C). Because the exact temporal appearance and duration of these phases varied in each animal, we pooled the data from temporally similar cases (Fig. 6C, *n* = 5). *In vitro*, similar phases have also been described in the CA1-region, with regard to postsynaptic membrane shifts, in the time course of theta-frequency stimulation [44].

We distinguished two periods in the initiation phase. During the first period we observed extreme fluctuations in granule cell excitability. A gradual drop in granule cell responsiveness marked the transition into the second period of the first phase (Fig. 6B, C). The occurrence of the subsequent burst, and the burst length, correlate with the duration of pre-burst neuronal silence [8].

We also subdivided the second, bursting phase into two periods (Fig. 6B, C, D). During the first period, dentate gyrus excitability underwent a fast augmentation, reflected by an increase in PS amplitude, and by the appearance of multiple spikes at a frequency of 150–200 Hz, which were accompanied by a negative shift of direct current (DC) potential (Fig. 6A). Sustained DC-potential shifts of the electroencephalogram (EEG) are typically associated with extracellular increases of potassium, as a result of prolonged depolarization of large neuronal populations [44], [45], [46]. The amplitude of the DC-potential shift can be expressed with the amplitude of the current sink, preceding the EPSP (pre-EPSP, Fig. 6E). The second period of the bursting phase was formed and characterized by increases, up to the maximal values, of the pre-EPSP current sink and of the positive field potential amplitude (PA), paralleled by a decrement of population spikes (Fig. 6D, E). Highly increased values of the PA that are characterized by a late peak (15 ms) and a prolonged latency are defined as voltage-dependent “giant” EPSPs known as large depolarizing potentials, whose appearance has been related to NMDA activation [47]. The induced bursting, and subsequent NMDA activation, appear to be the main factors that determine the degree of the subsequent synaptic strengthening. We consider this phase as a crucial element in the induction of synaptic plasticity under this stimulation protocol and thus subjected the fEPSP to further detailed analysis (see below). We don't claim that the epileptiform after-discharges during 8-Hz stimulation are a physiological phenomenon, but the advantage of this protocol, in comparison to the standard 100 Hz stimulation, is to amplify, on population levels, the impact of complex spikes that occur during natural theta epoch. This permits a better analysis of the relationship between the intrinsic spiking activity and the subsequently occurring LTP.

The third, termination, phase represents a gradual decrease of the granule cell responsiveness with a parallel rise of the pre-EPSP current source and is a result of a compensatory release of potassium from the astrocytic network, in response to intensive neuronal activity [48].

### Field bursting correlates with long-lasting modifications of fEPSP response

**Figure 7:**
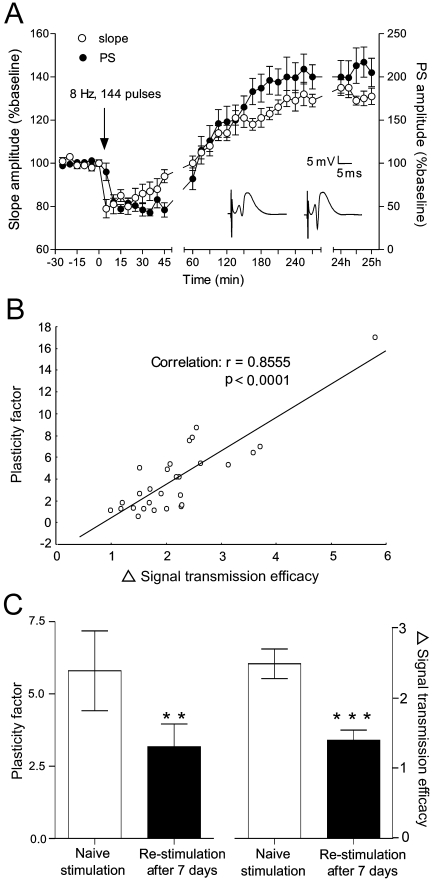
LTP correlates with field spiking activity. (A) fEPSP slope and PS amplitude show initial short-term depression and subsequent long-lasting potentiation after 8 Hz stimulation (ANOVA, *p*<0.001, *n* = 16). Analog traces represent fEPSPs, prior to and after stimulation, respectively. (B) Correlation between the change in signal transmission efficacy (ΔSTE) and 8 Hz stimulation-related plasticity factor (PF) in a group of naive animals (*n* = 28). (C) Comparison of PF and ΔSTE between a group of naïve animals that received stimulation, and a group of animals that received re-stimulation after 7 days, indicate a significant decrease of probabilities after re-stimulation (**, *p*<0.01, student's paired *t* test, *n* = 6).

The 8 Hz (144 pulse) stimulation protocol induced late-onset, stable synaptic and non-synaptic changes in the recorded population (Fig. 7A). The probability that a neuron will fire in response to a particular afferent stimulation increases with the degree of the synaptic potentiation, measured extracellularly by the initial slope of the fEPSP. In parallel, the modified neuronal excitability, a non-synaptic component of LTP, also influences the final response and is reflected by the PS amplitude. The mechanisms underlying this phenomenon comprise voltage-sensitive changes of intrinsic excitability and/or shift in net inhibition [49]. In summary, the change in the signal transmission efficacy (ΔSTE) can be described as a function of the fEPSP slope and the PS (see methods).

ΔSTE tightly correlated with plasticity factor (PF, see methods) values in control animals (Pearsons, *r* = 0.855; *p* = 0.0001, *n* = 28), hence demonstrating that test-pulse-evoked neuronal activity in the bursting phase was the reason for the subsequent LTP (Fig. 6B). Moreover, both parameters were concordantly decreased when the 8 Hz stimulation protocol was given 7 days later to the same animals (*n* = 6) (Fig. 6C), demonstrating a long-term regulation of the modified neuronal network. The single effect of DC shift-related pre-EPSP current sinks on ΔSTE shows no significant correlation (Pearsons, *r* = 0.063; *p* = 0.75, *n* = 28, data not shown), indicating that epileptiform activity *per se* is not a prerequisite for synaptic plasticity.

### Theta-induced field burst firing in the dentate gyrus requires the involvement of both NMDA receptors and voltage-gated Ca^2+^ channels

**Figure 8:**
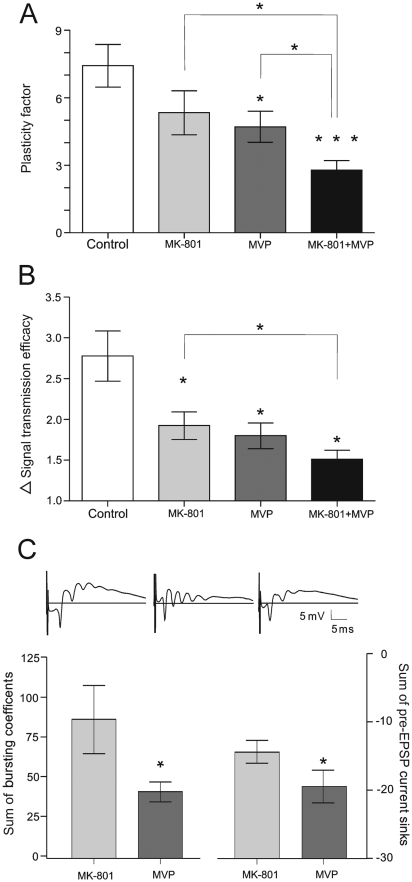
8 Hz-induced LTP depends on Ca^2+^ entry during the bursting phase. The NMDA receptor non-competitive antagonist, MK-801 (0,15 mg/kg i.p.; *n* = 6) and VGCC blocker Methoxyverapamil (MVP, 100 nmol/5 µl.i.c.v.; *n* = 6) reduce the plasticity factor (PF) values (A) (student's paired *t* test, *, *p*<0.05: indicates significance compared to the control group) as well as signal transmission efficacy (ΔSTE) values (B) (student's paired *t* test,*, *p*<0.05: indicates significance compared to the control group). (C) NMDA and VGCC components in the PF impairment. Upper analog traces: Examples of recording samples show impaired pre-fEPSP current sink and compensatory spiking activity in the MK-801-treated group (middle) compared with the control (left). The right trace reveals reduced polyspikes in the MVP group. Field bursting activity under MK-801 block is increased in comparison to VGCC antagonism (bottom left), and the reduced sum of pre-EPSP current sinks is related to impaired NMDA function (bottom right).

As NMDA receptors and voltage-gated Ca^2+^ channels (VGCC) comprise significant postsynaptic sources of Ca^2+^ during induction of LTP in the dentate gyrus in vivo [50], their antagonism should affect the potentiation probability, and thus the subsequent expression of LTP. The non-competitive NMDA receptor antagonist, MK-801 (0.15 mg/kg, i.p.), as well as L-type VGCC blocker methoxyverapamil (100 nmol/5 µl, i.c.v), partially abolished the plasticity factor (Fig. 8A) and subsequently ΔSTE (Fig. 8B). In the course of stimulation, the block of the NMDA receptor mainly decreased pre-EPSP current sinks, combined with increased field bursting activity (Fig. 8C) (student's paired *t* test, *p*<0.05, *n* = 10). This corresponds to the dependency of NMDA charge transfer on the firing duration [51]. The VGCC block (Fig. 8C) had the opposite effect, reducing the amplitude and the number of the population burst spikes (student's paired *t* test, *p*<0.05, *n* = 10). When both NMDA receptors and a fraction of VGCC were blocked together, a robust inhibition of LTP occurred (Fig. 8A, B), as was shown previously, in vitro, for CA1 distal synapses [37] and in vivo in the dentate gyrus [50].The residual increase of ΔSTE, can be accounted for by Ca^2+^-entry through other means, such as T-type VGCCs. These results are consistent with reports of a disruption of spatial learning after blockade of NMDA receptors and hippocampal LTP [52], [53], [54] and particularly when synaptic weights in dentate gyrus are affected (Brun *et al*., 2001; but see also [55], [56].

### Synaptic plasticity evoked by theta-induced field burst firing in the dentate gyrus is input-specific

**Figure 9:**
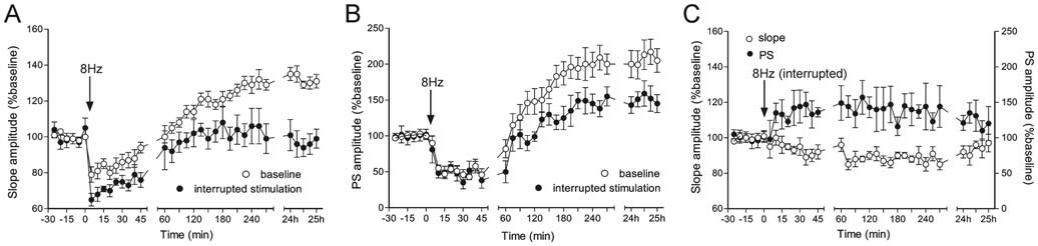
Dissociated test-pulse stimuli and field bursts result in a lack of fEPSP potentiation. (A) Comparison of the fEPSP slope between animals that experienced an interruption of 8 Hz stimulation prior to the bursting phase, and control animals (that received 144 pulses). Note the immediate difference after the stimulation, during the depression period (ANOVA, *p*<0.01, *n* = 6). (B) PS amplitude in the same groups. A non-specific significant increase in PS is observed in the non-paired group, when compared to the baseline period values (ANOVA, *p*<0.01, *n* = 6). (C) Modification of granule cell response after discontinued theta-like stimulation of the medial perforant path, in cases where no generalized field response occurred (*n* = 5). fEPSP and PS show opposite behaviour - depression and potentiation, respectively. This E-S uncoupling continues for about 24 h.

Our results demonstrate that synchronous network activity is sufficient to produce stable, long-term alterations in the strength of active afferent connections. Here, we investigated the effects of unpaired afferent activity with intrinsic field bursting, (with the same amplitude as for the LTP study) on the direction of synaptic plasticity. For this purpose we discontinued providing electrical pulses in the course of 8 Hz stimulation, once the late initiation phase was reached. Dissociating perforant path stimuli and intrinsic field discharges in the bursting phase, resulted in no potentiation of the fEPSP, compared to baseline values (Fig. 9A) (ANOVA, *p*>0.05, *n* = 6). Moreover, the difference between the paired and non-paired groups was evident immediately after the stimulation during the depression period. The intrinsic excitability represented by the PS amplitude was less expressed compared to the control group (Fig. 9B), but still was significantly augmented in comparison to baseline levels (ANOVA, *n* = 6, *p*<0.01). This result confirms that the neuronal discharging probability could be enhanced as a non-specific effect caused by continuous depolarization. Interestingly, in a few cases (38%) the interrupted 8 Hz stimulation elicited small amplitude field discharges, and population spikes that were insufficient to produce a termination phase. The EEG revealed a lack of negative shift of DC-potential and subsequently no postical depression period was observed. This group of animals comprises a control that excludes the side effects of generalized hyperexcitation, concerned with burst firing of large neuronal populations. The changes in fEPSP slope and PS occurred immediately, without a preceding depression period (*n* = 5; Fig. 9C). E-S uncoupling that was characterized by an enhancement of PS and a decrease of fEPSP occurred for a few hours after the stimulation (Fig. 9C). This result confirms the findings after brief 8 Hz and 2.5 Hz stimulation protocols where E-S uncoupling is a result of temporal dissociation between postsynaptic and presynaptic activity.

## Discussion

Our data describe the relationship between postsynaptic multiple spikes and the naturalistic induction of persistent LTP in the freely behaving rat. We report that long-term synaptic plasticity occurs in the dentate gyrus after phasic activation of entorhinal afferents in the theta-frequency range. This plasticity is proportional to the bursting ability of granule cells. LTP of the synaptic component only occurs upon temporal proximity between stimulation via the entorhinal input and increased dentate spiking activity. Effects are input-specific and require activation of both NMDA receptors and voltage-gated calcium channels. Taken together, our findings support that dentate gyrus granule cells change their firing patterns proportionally to the preceding spiking activity of afferent inputs and that synaptic plasticity occurs as a result of this.

The dentate gyrus is an important component of the successful acquisition of spatial information. Rats with dentate gyrus lesions show impaired learning in the Morris water maze [57], [58], [59] and are also impaired in the acquisition of spatial contextual fear conditioning [60]. Episodic memory formation is characterized by the appearance of theta oscillations in the limbic system (Lisman, 1999; Buzsaki, 2002; Buzsaki and Draguhn, 2004). The temporal features of theta oscillations are thus believed to enable the assembly and segregation of neuronal groups in the hippocampus and associated structures, such that information is stored. Entorhinal cortex activity, particularly in the 5–15 Hz range, maximally enhances the polysynaptic excitation of hippocampal pyramidal neurons via the dentate gyrus [61]. The cyclic variation of excitation and inhibition during theta provides temporal windows of opportunity to selectively ignore or enhance the effectiveness of presynaptic activity [7]. Synchronized depolarization characterizes theta rhythm in the limbic system where each theta cycle is proposed to serve as an information quantum [1], [62]. This field depolarization involves single spikes [4] or complex spike bursts [6] of the place cells that fire according to the animal's spatial position. Multiunit recordings from the CA1 region have shown that compared to single spikes, bursts of spikes lead to a significantly increased synaptic effectiveness, which positively correlates with burst length [7], [8]. Our data support and develop these findings. Analysis of the effects of amplifying naturally-occurring single cell plasticity, on a population level, demonstrates that long-lasting changes of the fEPSP occur only in animals where a robust increase in the spectral power of theta range was evident. The EEG of these animals displayed multiple spiking events superimposed on the positive phases of theta. We found also a highly-significant correlation between field spiking activity and fEPSP plasticity.

Our findings demonstrate that stimulation-induced theta-related plasticity had an opposite effect on granule cell excitability and synaptic responses. The two components of LTP distinguished through extracellular recordings are (1) synaptic, measured by changes in the fEPSP slope and (2) excitatory, measured by the changes in the population spike [31], [34], [49]. Potentiation of the population spike is often correlated with the triggering of spike discharges at stimulus strengths previously subthreshold for spike initiation [31]. EPSP-Spike plasticity or E-S plasticity comprises an increase in the probability that an fEPSP will elicit an action potential [42]. The mechanisms underlying E–S potentiation are believed to include a decrease in the ratio of inhibitory to excitatory drive [34], [63], [64] and/or an increase in the intrinsic excitability of the postsynaptic neuron through modulation of postsynaptic voltage-gated conductances [65], [66], [67]. Whether the synaptic component of LTP undergoes potentiation or depression depends on the temporal proximity between pre- and postsynaptic activation. Superimposed on augmented theta oscillations, the test-stimuli gain power and can induce potentiation [12], [13]. When coincided with the trough of theta, the test-stimuli will evoke depression [18]. The timing corresponds ideally with synchronized depolarization during theta oscillations, where the theta cycle is regarded as an information quantum.

LTP is more effectively induced in the dentate gyrus in vivo when the tetanus is delivered on positive phases of theta [13]. In line with these studies, our data show that in cases where test-pulse stimulation coincides with the positive peak of theta, the fEPSP is increased in comparison with cases where these events are separated in time. We used 2.5 Hz stimulation to test this further, because it falls randomly upon the theta cycle (the frequency of which was about 8 Hz in our animals). We observed that this stimulus-theta interplay is ineffective in potentiating the fEPSP slope. The synaptic depression which was observed rather derived from the application of 900 pulses at this low-frequency. The depression of the fEPSP seen may also reflect the occurrence of heterosynaptic LTD, which results when an inactive input is combined with cellular depolarization during theta episodes [41]. This leads to no change in ΔSTE, and contributes to the enhancement of the signal-to-noise ratio during the pattern separation of active afferents that carry new information. Physiologically similar local modifications have been shown in the form of a slight decrease in fEPSP, after behavioural conditioning [68]. In our case the “test-pulse” input was predominantly inactive or “silent” during the local theta-associated activity, and soon after the stimulation it underwent depression. Similarly, we demonstrate that dissociating the entorhinal test-pulse and the endogenous synchronous activity, in cases where generalized excitation after 8 Hz, 144 pulses stimulation occurred, does not lead to potentiation of the fEPSP. Thus, when the stimulation protocol discontinued between the initiation phase and intrinsic polyspiking activity, thereby causing dissociation between the afterdischarges and the afferent test-pulses, no EPSP potentiation occurred. A significant increase of neuronal excitability remained nonetheless, confirming that the side-effect of large synchronized activity is associated with non-specific, long-term enhancements of the PS amplitude.

Both 8 Hz and 2.5 Hz stimulation protocols resulted in a shift of the peak theta frequency. The peak theta values shifted from 7–8 Hz, before stimulation, towards 10–12 Hz after stimulation, e.g. from lower to higher ranges of theta. While the lower theta component is atropine-sensitive [14], and is mediated by the medial septum, the higher component of hippocampal theta is conveyed by entorhinal afferents to dentate gyrus neurons [3], [69]. Synaptic plasticity induced through stimulation of the perforant pathway may tune the intrinsic properties of cell-interneuron feedback [70], [71], resulting in oscillations at slightly different frequencies, and phase, than the initial theta rhythm [69]. In line with this, we observed a theta shift due to 8 Hz (30 pulse) stimulation, and due to 2.5 Hz (900 pulse) stimulation, in those animals that expressed synaptic plasticity (e.g. the E-S uncoupled groups). For the 8 Hz,144 pulse protocol, the intrinsic properties of the dentate gyrus were ‘phase-locked’ at 8 Hz in the course of the protocol, as indicated by the appearance of robust spiking activity only after perforant path pulses, which in turn prevented a phase drift during the stimulation. These findings confirm the dominant role of the perforant input in the regulation of the higher theta rhythm [3].

Besides the precise timing of the pre- and postsynaptic activities, the other major factor determining changes in synaptic strength comprises the frequency of the postsynaptic response. One of the more convincing links between the frequency of neuronal discharges and hippocampal LTP involves the use of theta-frequency stimulation [72], [73], [74], [75]. This stimulation protocol is patterned after the endogenous theta rhythm, and is known to induce effectively LTP when short 100 Hz bursts are delivered at 5 to 8 cycles per second. Bursts of spikes, observed during theta rhythm in vivo, also comprise a natural stimulus for inducing LTD [20]. We examined how polyspiking activity on a population level relates to the phenomenon of LTP in the freely behaving rat. Multiple spiking activities were evoked by phasic activation of entorhinal afferents at 8 Hz: a theta-range frequency that we had observed when our animals engaged in novel exploratory activity. By analysing the field bursting EPSP parameters (including the amplitude, number and duration of the population spikes), we established that the long-term increase of entorhino-hippocampal synaptic strength is proportional to the preceding spiking activity. We induced field discharges with theta frequency and examined the effect on subsequent synaptic plasticity, based on the premise that a temporal coordination of presynaptic and postsynaptic activities may be a necessary condition for the induction of LTP [1], [76]. Furthermore, we showed that the relative change of the fEPSP and the increased signal transmission efficacy in dentate region after 8 Hz stimulation depend exactly on the degree of bursting-evoked Ca^2+^ influx.

These data offer a mechanism by which network activity in the theta frequency leads to synaptic plasticity in the hippocampus, explains how integration of network activity with synaptic plasticity may enable hippocampal information storage [77], [78]. Our findings are consistent with synchronization-dependent memory models, and offer an explanation as to how frequency-dependent patterns of network activity lead to long-term changes of synaptic weight.

### Concluding comments

The major findings of the present experiments are that (1) stimulation-induced theta oscillation elicits synaptic plasticity in the dentate gyrus; (2) bursting activity in the course of theta rhythm correlates with the degree of the subsequent long-term synaptic and excitatory changes; (3) voltage-gated Ca^2+^ channels, in combination with NMDA currents, mediate the burst-associated induction of long-lasting synaptic potentiation *in vivo*; (4) the change in the firing probability caused by the specific input depends on the temporal proximity between presynaptic activity and postsynaptic burst discharge; (5) when these events are temporally non-correlated a theta-dependent E-S uncoupling occurs.

## Materials and Methods

### Ethical approval

Stereotaxic surgery procedures, and all animal experimentation described here, were carried out in accordance with the regulations set down by the Federal Republic of Germany, and were approved by the local authorities (Bezirksregierung Arnsberg).

### Electrode Implantation

Under sodium pentobarbitone anaesthesia (Nembutal, 40 mg/kg, i.p., Serva, Germany), male Long Evans rats (7–8 weeks old) underwent electrode implantation into the dentate gyrus as previously described (Manahan-Vaughan et al, 1998). A cannula was also permanently implanted into the ipsilateral cerebral ventricle to enable drug injections. The animals were allowed 7–10 days to recover from surgery before extracellular recordings in freely behaving animals were commenced.

### Experimental set-up

The head-stages of the rats were connected by a ribbon cable via a swivel connector to the experimental set-up. The cable allowed the rats to move freely in the recording box. Evoked responses were generated by single biphasic square wave pulses of 0.2 ms duration and a manually-specified stimulation intensity of 100 µA–900 µA using a constant current stimulator unit (A385R, World Precision Instruments, USA). The signals from the recording electrodes were amplified using an AC-coupled A-M-Systems 1700 differential amplifier (100x), filtered (0,1 Hz–10 KHz bandpass) and digitised at 10 KHz through a DA/AD converter (CED 1401-plus, Cambridge Electronic Design, UK). Waveforms were displayed and analysed on a PC with data acquisition software (Pwin, Leibniz-Institute Magdeburg).

### Measurement of Evoked Potentials

Responses were evoked by giving test-pulses (0.025 Hz, 0.2 ms stimulus duration, 16 000 Hz sample rate). For each time-point, five evoked responses were averaged. Both field excitatory post-synaptic potential (fEPSP) slope and population spike (PS) amplitude were monitored. The amplitude of PS was measured from the peak of the first positive deflection of the evoked potential to the peak of the following negative potential. fEPSP slope was measured as the maximal slope through the five steepest points obtained on the first positive deflection of the potential (for the stimulation protocol recordings it was considered as a ratio of the fEPSP amplitude and fEPSP latency). By means of input–output curve determination, the maximum PS amplitude was found for each individual animal and all potentials employed as baseline criteria were evoked at a stimulus intensity which produced 40 % of this maximum. Synaptic plasticity was induced by stimulation protocols with frequency of 2.5 and 8 Hz. The 2.5 protocol consisted of 900 pulses, except for the cases with an interrupted stimulation, while the 8 Hz stimulation protocol consisted of 30 pulses or 144 pulses. In 40% of all cases, we observed a generalized dentate gyrus response to 2.5, comprising of field discharges during the stimulation period, while for 60% of all cases, generalized activity was absent. For the groups of animals where stimulation protocols were interrupted, visual monitoring of the fEPSP changes and EEG in the course of stimulation was used to determine the exact time point of pulse cessation for each animal. The stimulus amplitude for this protocol was the same as that used for recordings. Typical exploratory behaviour of the animals was observed for a few minutes after the 8 Hz stimulation. EEG was monitored for 4 h after the stimulation and no epileptiform periods were detected. Statistical significance was estimated by using (between-factor) ANOVA with repeated measures and by post hoc Student's *t* tests (*P*<0.05 was taken as indicating statistical significance).

### Measurement of computed parameters

The field excitatory postsynaptic potential (fEPSP) slope and the population spike (PS) amplitude were recorded and analysed. The fEPSP was used as a measure of postsynaptic depolarisation. The PS reflects the somatic response, the amplitude of which is dependent on the number of granule cells that discharge in synchrony. The potentiation of the PS is often correlated with the triggering of spike discharges at stimulus strengths, previously subthreshold for spike initiation [31]. These two components (fEPSP slope, PS) of LTP comprise independent processes [31], [32], [33], [34]. The product of the synaptic component of LTP (fEPSP) together with the non-synaptic component (PS) has a much stronger effect on the neuronal output in comparison to cases when only one of these two components is potentiated. This combined product reflects an enhancement of the signal-to-noise ratio with regard to signal transmission along neuronal networks [35]. To evaluate this possibility, we assessed the change in signal transmission efficacy (ΔSTE), and described this as a function of synaptic and excitatory potentiation ratios:
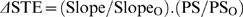
where Slope (of the fEPSP) and PS comprise the mean values of the recorded potentials in the 4^th^ and 24^th^ hour after the theta-like stimulation. Slope_o_ and PS_o_ represent, on the other hand, the baseline recorded mean values.

The EPSP-to-spike (E-S) ratio was derived from the mean of the PS/Slope ratios, obtained for a period of 4 h after the stimulation protocol (from the 7^th^ till the 30^th^ recording measurement).

### Factors determining potentiation probability

The probability of synaptic potentiation (as a result of polyspiking activity) is determined by the following factors: (1) the bursting coefficient, (2) the pre-EPSP current sink and (3) positive field potential amplitude (PA) (Fig. 6D, E). The bursting coefficient (BC) represents the spiking activity during the burst:

The bursting coefficient (BC) represents the spiking activity during the burst: 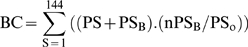
where PS represents the population spike amplitude recorded during the stimulation; PS_B_ is the amplitude of the additional burst spikes and nPS_B_ is their number; PS_o_ reflects population spike amplitude recorded during the baseline period. Soma-dendritic backpropagation of action potentials comprises a mechanism through which bursts contribute to synaptic plasticity [1], [36]. Backpropagation spikes, together with coincident synaptic depolarization, evoke a massive Ca^2+^ influx [10], which in turn, triggers the intracellular signalling pathways of LTP. A strong correlation exists between the amount of somatic depolarization and the magnitude of LTP [37].

The NMDA receptor complex is known to be a coincidence detector of two factors: ligand and voltage; where the coincidence detection is represented by a different amount of Ca^2+^ influx [38]. The burst length (number of burst spikes) correlates to the extracellular spike amplitude and to the rising slope of the intracellular action potential [8]. Bursts may function as “conditional synchrony detectors” that determine the outcome of synaptic plasticity [1], [8]. Therefore, we considered the parameter that determines the length of the burst (number of spikes), to be an important factor leading to synaptic plasticity, and which therefore must comprise part of the bursting coefficient equation.

The degree of extracellular-measured membrane depolarization is related to pre-EPSP current sinks. Although they are the result of non-physiological epileptiform field hyperexcitability, we used them as a model to amplify single depolarization events that occur during theta epochs. Hence, the next factor that we considered is the development of the potential amplitude (PA). This is an additional mechanism that leads to prolonged Ca^2+^ influx due to a voltage-dependent reduction of the Mg^2+^ block of NMDA receptors. In summary, these parameters can be united in an equation that determines the possible plasticity effect on the neurons, represented by the plasticity factor (PF): 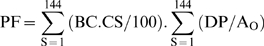
in which Σ(BP.CS) represents the sum of all values, that are product of the BC and the pre-EPSP current sink (CS) at each point in the time course of 8 Hz (144 pulse) stimulation. Σ(PA/PA_o_) reflects the ratio between the amplitude of the positive field potential and the mean amplitude of the fEPSPs recorded prior to the stimulation. The PF defines an activity-dependent possibility for a long-term increase or decrease of the population spike and/or fEPSP slope.

### Data Analysis of network activity

The intrahippocampal electroencephalogram (EEG) was obtained via recordings from the granule cell layer of the dentate gyrus. The EEG was sampled at 0.5 kHz and stored on hard disc for further off-line analysis, in order to evaluate delta (1–3,5 Hz), theta (4–10 Hz), alpha (10–13 Hz), beta1 (13,5–18 Hz), beta2 (18,5–30 Hz) and gamma (30–100 Hz) oscillatory activity during the course of experiment. Fourier analysis of artefact-free epochs was performed with the Hanning window function using “Spike2” software (Cambridge Electronic Design). The absolute values of spectral power for each individual animal were transformed into relative ones (with the mean value for baseline pre-injection period taken as 100%) that were used for statistical analysis. For each time point, the results of Fourier analysis of five epochs were averaged. The 2.5 Hz stimulation data were analyzed by off-line spectral power measurements of the interstimuli EEG epochs. For each EEG epoch, the data of spectral analysis represent the values of spectral power in all frequency windows. The range of these frequency windows was determined by the stimulation protocol parameters, which allowed recordings of EEG epochs with duration of 250 ms. This comprised an uncontaminated EEG epoch that occurred between two artificial test-spikes at a frequency of 2.5 Hz. The statistical treatment and analysis of data, included the calculation of descriptive statistics (mean, S.E.M.), and analysis of variance (ANOVA). For the correlation analyses we used Pearson's coefficient of comparison.

### Compounds and Drug Treatment

The L-type voltage-gated Ca^2+^ channel (VGCC) blocker methoxyverapamil (MVP) (Biotrend Chemikalien GmbH, Köln, Germany) was dissolved in H_2_O and prepared in concentration of 100 nmol/5 µl. MVP or vehicle were injected, into the lateral cerebral ventricle, in a 5 µl volume over a 6 min period via a Hamilton syringe. The Hamilton syringe was connected by means of a flexible polyurethane tube to an injection cannula which was inserted into the permanently implanted cannula. Antagonist or vehicle injection was carried out 30 min prior to stimulation to enable diffusion from the lateral cerebral ventricle to the hippocampus to occur (Manahan-Vaughan et al, 1998). The non-competitive NMDA receptor antagonist, MK-801 (Biotrend Chemikalien GmbH, Köln, Germany) was dissolved in 0.9% NaCl and applied by intraperitoneal injection in concentration of 0.15 mg/kg, i.p. The concentration we used is much lower than the concentrations of MK801 (e.g. 5 mg/kg, i.p.) known to induce behavioural and locomotor alterations [39] and and in the range of MK801 concentrations (e.g. 0.05 mg), that are known to impair the acquisition of a spatial memory task without affecting locomotor activity [40].
